# Patterns of *spon1b:GFP* expression during early zebrafish brain development

**DOI:** 10.1186/s13104-019-4876-x

**Published:** 2020-01-07

**Authors:** Nathalie Agudelo-Dueñas, Manu Forero-Shelton, Irina V. Zhdanova, Veronica Akle

**Affiliations:** 1 Biophysics Group, Department of Physics, Universidad de los Andes, Bogotá, Colombia; 2 Neuroscience and Circadian Rhythms Laboratory, School of Medicine, Universidad de los Andes, Bogotá, Colombia; 3 BioChron LLC, Boston, MA USA

**Keywords:** F-spondin/*spon1b*, GFP, Light sheet fluorescence microscopy (LSFM), Zebrafish (*Danio rerio*), Olfactory system, Pituitary/hypophysis, Habenula

## Abstract

**Objective:**

F-spondin is part of a group of evolutionarily conserved extracellular matrix proteins in vertebrates. It is highly expressed in the embryonic floor plate, and it can bind to the ECM and promote neuronal outgrowth. A characterization of F-spondin expression patterns in the adult zebrafish brain was previously reported by our group. However, given its importance during development, we aimed to obtain a detailed description of green fluorescent protein (GFP) expression driven by the *spon1b* promotor, in the developing zebrafish brain of the transgenic *Tg(spon1b:GFP)* line, using light sheet fluorescence microscopy (LSFM).

**Results:**

Images obtained in live embryos from 22 to 96 h post fertilization confirmed our earlier reports on the presence of *spon1b:GFP* expressing cells in the telencephalon and diencephalon (olfactory bulbs, habenula, optic tectum, nuclei of the medial longitudinal fasciculus), and revealed new *spon1b:GFP* populations in the pituitary anlage, dorso-rostral cluster, and ventro-rostral cluster. LSFM made it possible to follow the dynamics of cellular migration patterns during development.

**Conclusions:**

*spon1b:GFP* larval expression patterns starts in early development in specific neuronal structures of the developing brain associated with sensory-motor modulation. LSFM evaluation of the transgenic *Tg(spon1b:GFP)* line provides an effective approach to characterize GFP expression patterns in vivo.

## Introduction

Spondins are a family of evolutionarily well-conserved extracellular matrix proteins characterized by the presence of thrombospondin domains. Studies of F-spondin have shown that this protein enhances neurite outgrowth, promotes nerve precursor differentiation [1] and acts as an adhesion and axon guidance molecule [2].

In zebrafish, *spon1b* is expressed in the forebrain, midbrain and hindbrain regions [3]. In our previous work [4] using the transgenic *Tg(spon1b:GFP)* line, we reported F-spondin expression in the brain and eye regions as early as 18 h post-fertilization (hpf); in particular, in the notochord, floorplate, and flexural organ, in neurons extending long neuronal tracks in the CNS, and in peripheral tissues with active patterning or proliferation throughout development. A general description of *spon1b:GFP* expression patterns in the transgenic *Tg(spon1b:GFP)* line was done both in zebrafish embryos and adults [4]. A detailed characterization of the GFP expression driven by the *spon1b* promotor in zebrafish embryos would further contribute to our understanding of the roles this protein plays during early vertebrate development.

Light sheet fluorescence microscopy (LSFM) allows imaging individual embryos at high resolution in three dimensions over time due to reduced phototoxicity. We can resolve individual cells of single individuals over periods of 24 h using LSFM; thus, by monitoring fluorescence we were able to determine the initial expression and the dynamics of *spon1b:GFP* positive cells within each brain structure. We tracked GFP positive cell populations starting at 22 hpf, up until 4 days post-fertilization (dpf), and observed that GFP is initially expressed in specific clusters of cells in the dorsal and ventral portions of the developing telencephalon and diencephalon.

## Main text

### Results and discussion

#### *spon1b:GFP* expressing cell populations between 22 and 96 hpf

**Fig. 1:**
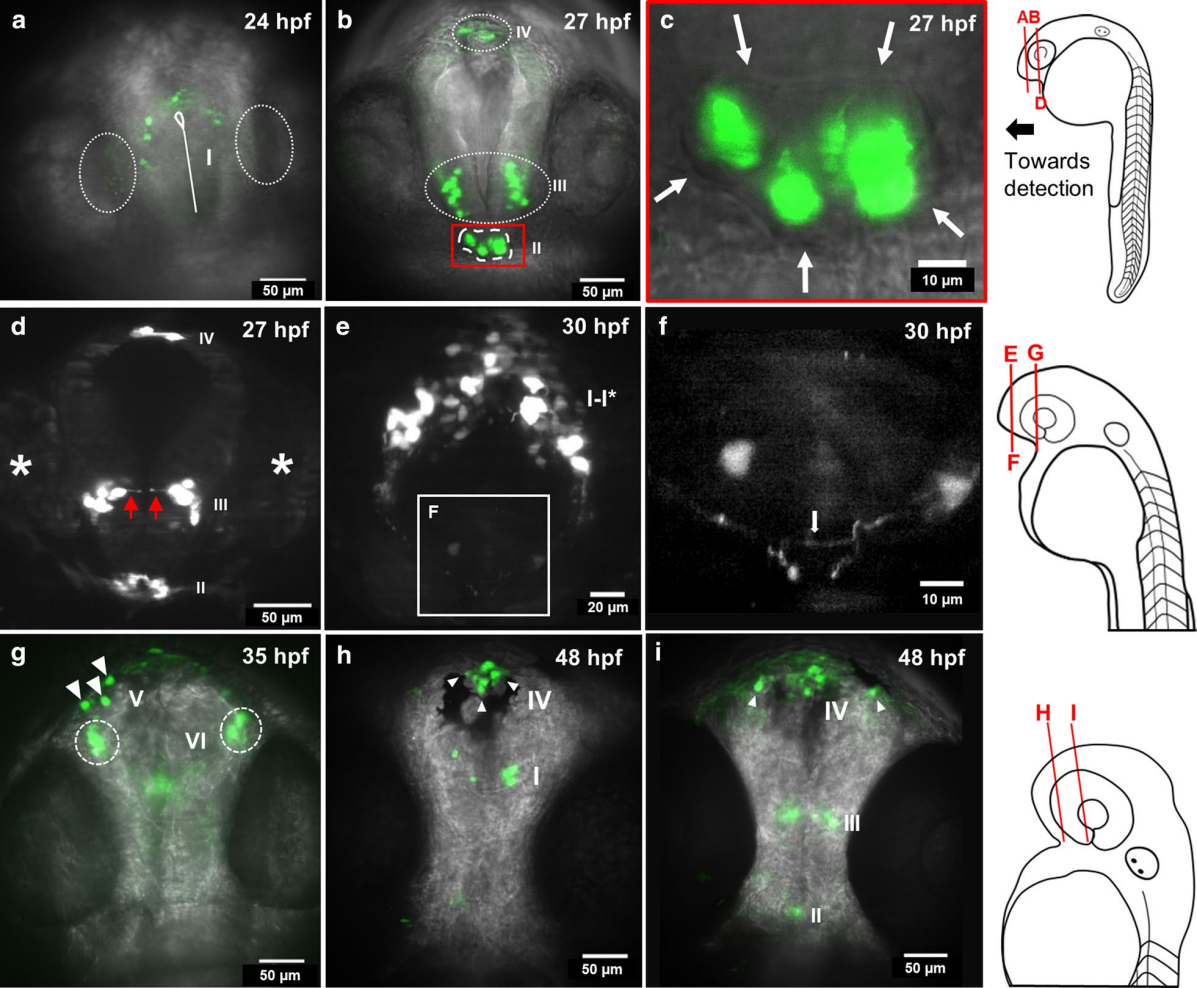
*spon1b:GFP* expression in cell populations from 24 to 48 hpf in telencephalic and diencephalic regions. **a** Maximum intensity projection (MIP) of a 10 µm optical slice of GFP fluorescence (green) overlaid with a transmitted light image (gray) for anatomical reference. This MIP at the dorsal telencephalon shows population **i**. Olfactory placodes are circled for anatomical reference. Telencephalic ventricle is shown with a continuous white line. **b** MIP obtained from a 30 µm thick slice at the developing dorsal and ventral telencephalon and diencephalon, showing populations II, III and IV. Population II corresponds to the pituitary anlage (dashed line), identified adjacent to the ventral diencephalon using transmitted light images as anatomical reference. **c** Detail of the pituitary anlage enclosed in red in (**b**). Arrows outline the border of the pituitary anlage. **d** Cells in population III show a characteristic morphology along the neuroepithelium. The MIP of a 12 µm thick slice of cells in population III of a different individual shows prolongations along the developing neuroepithelium at 27 hpf (red arrows), but at the same approximate location as (**b**). White asterisks indicate eye position. **e** MIP obtained from a 60 µm slice showing the tips of axonal processes in the midline and commissure at the telencephalon from populations I–I*. **f** Detail of axonal processes and commissure (white arrow) enclosed in (**e)**. **g** MIP obtained from a 75 µm slice at the dorsal diencephalon and tectum showing individual cells in newly identified population V (white arrowheads), and two bilateral clusters as VI (dashed circles). **h** MIP obtained from a 50 µm slice showing population I and IV (white arrowheads). **i** MIP obtained from a 90 µm slice showing populations II-IV. White arrowheads show cells from the olfactory system. Images from **a**–**i** are frontal views. Schematic drawings of zebrafish embryos at the right show the approximate position of planes in **a**–**i**

Cell populations expressing *spon1b:GFP* were monitored in the developing zebrafish brain of the transgenic *Tg(spon1b:GFP)* line starting at 22 hpf using LSFM. Between 22 and 24 hpf, *spon1b:GFP* expression is mainly observed in telencephalic and diencephalic regions, in four distinct populations identified here by roman numerals: I, II, III and IV (Fig. 1). Population I is the first identifiable cluster, surrounding the ventricle in a horseshoe pattern at the dorsal telencephalon (Fig. 1a).

From 25 to 36 hpf, new *spon1b:GFP* positive cells appear and increase the number of axonal projections among themselves (Fig. 1e). Their axons also project ventrally, toward the telencephalic midline, ending there or crossing the midline, while forming a commissure (Fig. 1e, f). Consistent with earlier neuroanatomical classifications, we identified cell population I as part of the telencephalic dorso-rostral cluster [5–7]. We suggest that these cells are part of the developing olfactory complex, consistent with the high expression in the olfactory bulbs of adults [4], and that some of the *spon1b:GFP* positive cells extend their axons contralaterally though the anterior commissure, as previously reported in zebrafish [7–9], and in rodents [10]. This observation was also supported by *spon1b:GFP* expression by cell clusters in the telencephalon at later developmental stages (48 hpf), which anatomically correspond to the olfactory epithelium and olfactory bulbs [11].

Cells in populations II, III and IV are in the same dorso-ventral plane, caudal with respect to population I (Fig. 1b). GFP positive cells corresponding to population II are within the developing hypophysis or pituitary anlage [12], surrounded by GFP negative cells, as confirmed by the overlay of transmitted and fluorescence light images (Fig. 1b, c). Population III is composed of bilateral symmetrically located cell clusters (Fig. 1b). These cells are distributed in the neuroepithelium, showing extensions toward the midline (Fig. 1d), and caudally projecting axons (data not shown). The number of cells in population III remains similar from 24 to 48 hpf (8–10 cells). Following the previous anatomical classification [5–7], we identified population III as the diencephalic ventro-rostral cluster. About five large cells (~ 10 μm in diameter) constitute population IV (Fig. 1b), which is located at the most rostral tip of the forebrain in a region similar to the one reported for the subcommissural organ [3]. These cells do not show significant changes up to 48 hpf. Further examination until 96 hpf confirms our previous studies, in which we did not detect *spon1b* in the developing subcommissural organ [4], because the area below the posterior commissure appears GFP negative. It is possible that the previous accounts of subcommissural organ by Higashijima et al. [3] were related to population IV described therein.

Starting at 28 hpf, two new *spon1b:GFP* populations appear in the dorsal diencephalon, named V and VI (Fig. 1g). Cells in V are larger than those in population VI, and are clearly separate from each other (Fig. 1g). Population VI appears around 31 hpf as two densely packed symmetric bilateral clusters (Fig. 1g). These two populations correspond to early expression in the habenula (Hb), consistent with other markers of habenular complex development with onset at ~ 32 hpf [13].

**Fig. 2:**
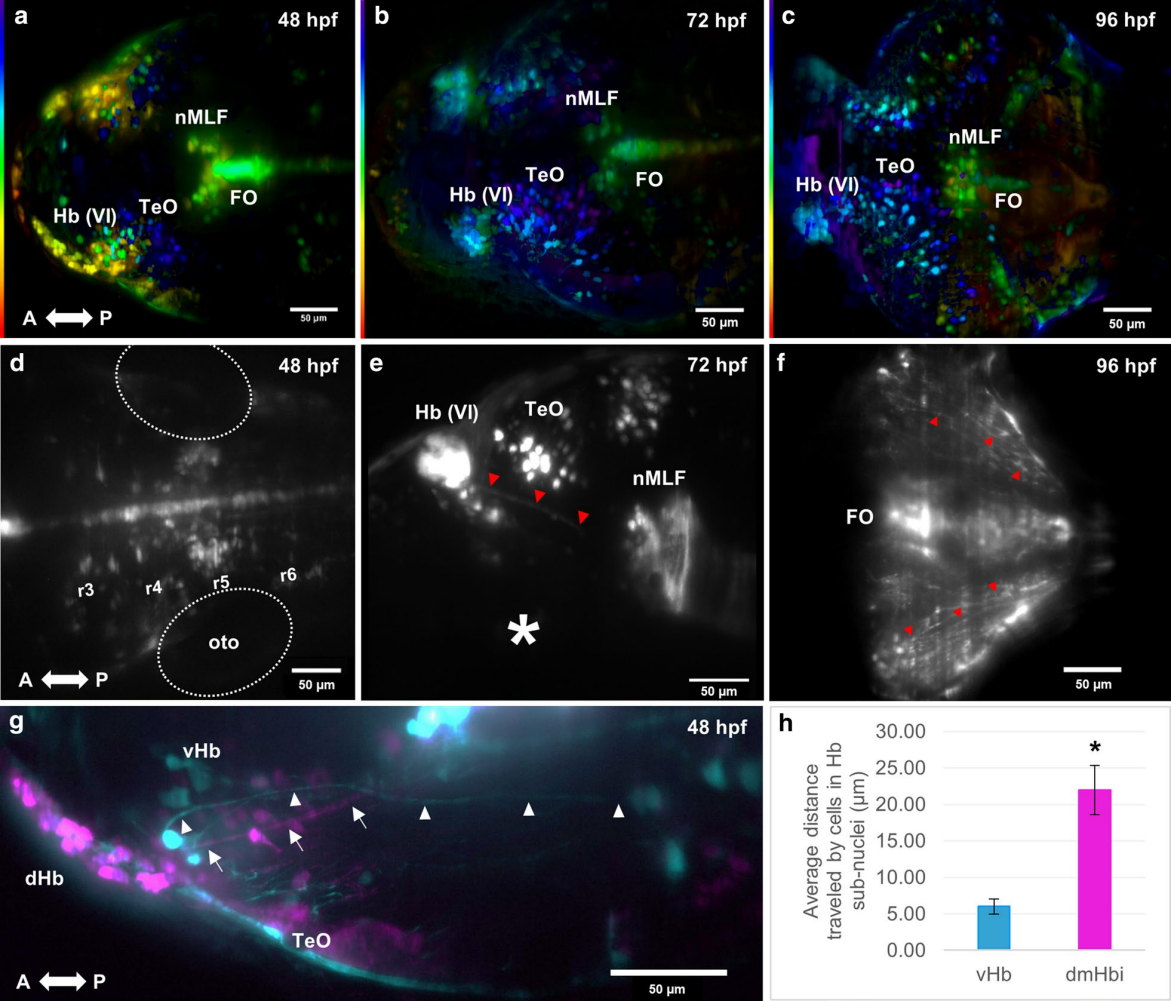
*spon1b:GFP* expression in cell populations from 48 to 96 hpf in telencephalic, diencephalic and hindbrain regions. Abbreviations: Hb, Habenula, dHb, dorsal habenula, TeO, Optic tectum, nMLF, Nuclei of the Medial Longitudinal Fasciculus, FO, Flexural Organ, a, Anterior, P, Posterior. **a** MIP color coded for a depth of 250 µm at 48 hpf. Cells in blue are part of the TeO. Cells in yellow are part of the developing Hb complex. Gamma was adjusted to a value of 0.75. **b** MIP color coded for a depth of 200 µm at 72 hpf. Cells in dark blue are part of the TeO. Cells in light blue are part of the developing Hb complex. Gamma was adjusted to a value of 0.75. **c** MIP color coded for a depth of 250 µm at 96 hpf. Gamma was adjusted to a value of 0.75. **d** MIP obtained from a 60 µm slice showing *spon1b:GFP* neurons in the hindbrain at 48 hpf. Rhombomeres (r3–6) are estimated by the position relative to the otocyst (oto). **e** MIP obtained from a 75 µm slice showing the Hb and the fasciculus retroflexus (red arrowheads) at 72 hpf. White asterisks indicate eye position. **f** Single plane showing an increased innervation at 96 hpf (red arrowheads). **g** Composite image of two MIPs obtained from a depth of 5 µm (cyan, depicting the vHb) and 20 µm (magenta depicting the dHb) at 48 hpf. Axons from the developing dHb are observed to project caudally, neighboring the nMLF (white arrows). Axons from the developing vHb project more caudally (white arrowheads), compared to axons from the dHb. Note the axons present within the tectal region. **h** Graph showing the average distance traveled by cells in the Hb subnuclei. The total distance traveled is significantly different (Mann–Whitney test, P value 0.0061) between cells in the dHb and the vHb subnuclei.. **a**–**d**, **f**–**g** are dorsal views. **e** is a lateral view

By 48 hpf, strong *spon1b:GFP* fluorescence expression appears in single cells of the optic tectum, and in individual motor neurons and projections of the nuclei of the medial longitudinal fasciculus (Fig. 1a). Between 72 and 96 hpf, the number of cells in the optic tectum increases, and different cell types are observed, which exhibit greater arborization, with axons projecting toward the tectal neuropil layers (Fig. 2b, c). This characteristic laminar structure of the optic tectum highlighted by *spon1b:GFP* positive cells and projections was well established at 96 hpf. Cells belonging to the flexural organ, first seen at 30 hpf, increase their fluorescence levels of *spon1b:GFP* to very high levels after 48 hpf (Fig. 2a).

At 96 hpf, there was also an increased innervation of the cerebellum and hindbrain with GFP positive projections, although no *spon1b:GFP* expressing neurons were detected in this area (Fig. 2f). It is possible that these axons constitute part of the visual circuitry, as previous studies describe connections between the tectum and nMLF [14] and the hindbrain [15] in zebrafish. In the hindbrain, *spon1b:GFP* positive neurons are located in rhombomeres 3 to 6 (r3–6) (Fig. 2d), as estimated by the position relative to the otocyst [16].

#### *spon1b:GFP* expression in the habenular complex

**Fig. 3:**
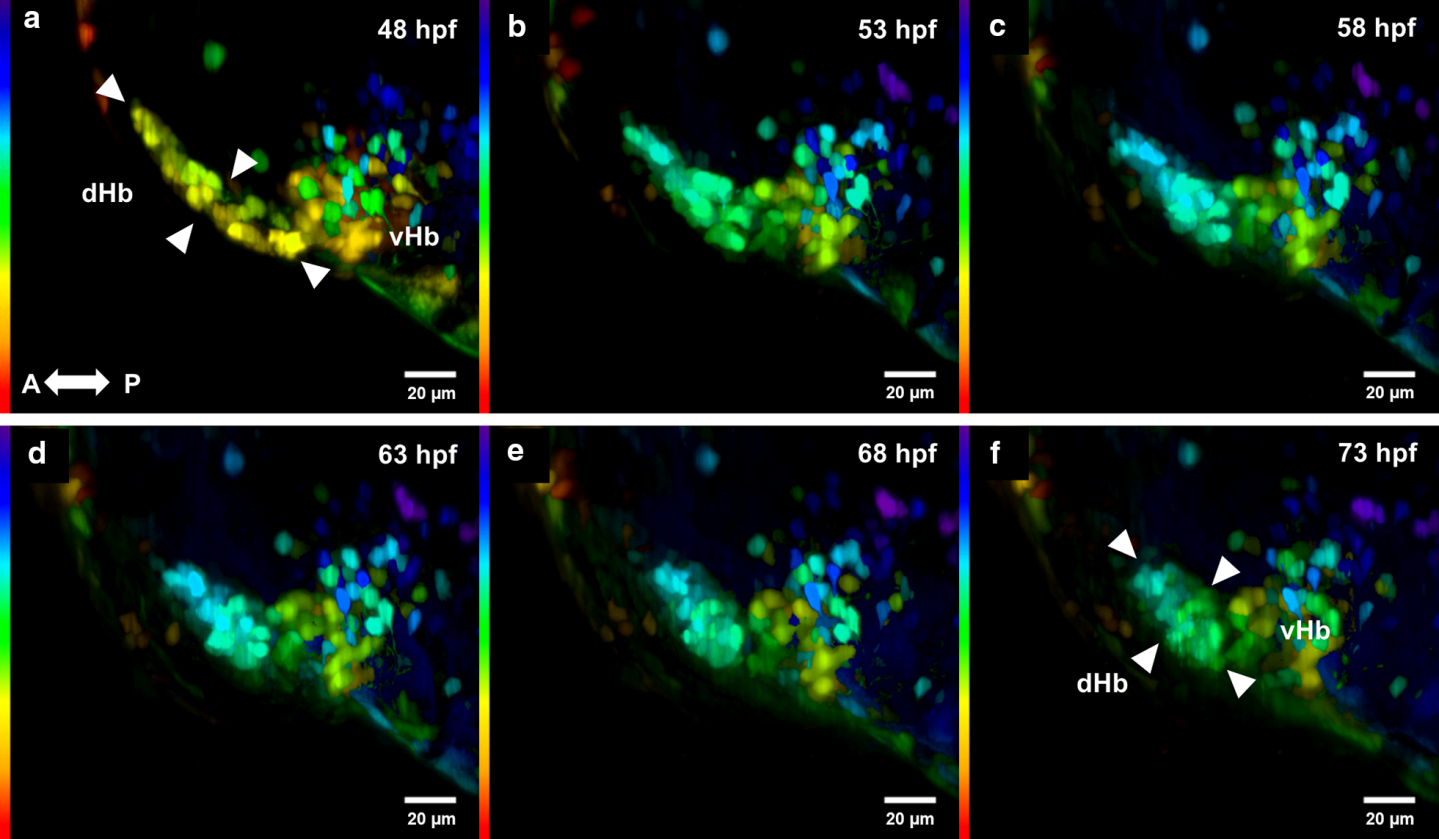
Development of the habenula from 48 to 73 hpf. Development of the Hb complex followed through time-lapse imaging. Cells within the dorsal habenula (dHb) aggregate progressively during development from an elongated shape to form a nucleus as indicated by the white arrowheads. The dHb is observed to be located more dorsally with respect to the vHb. Images from **a**–**f** are MIP color coded for a depth of 250 µm, purple and blue being more dorsal, and red and orange more ventral. Original stacks were cropped and aligned using the FiJi plugin Linear Stack Alignment with SIFT [20]. Gamma was adjusted to a value of 0.75. All time points are dorsal views. A, anterior, P, posterior

The difference in cell size between populations V and VI continues throughout development. These two populations remain separated through development, and are presumed to be ventral (vHb) and dorsomedial inferior habenula (dmHbi) subnuclei, respectively (Fig. 3). The dmHbi is part of the dorsal habenula (dHb). Between 48 and 72 hpf, detailed time-lapse tracking of populations V and VI in the same embryo revealed that these two subnuclei changed their relative position, with a close starting position and a final distance between the centers of ~ 14 µm. *spon1b:GFP* expressing cells in the dmHbi subnuclei progressively change from a long and extended string-like nucleus, to become a dense round cluster of cells (Fig. 3a, f). During the 24 h tracking, cells located at the anterior end of the dmHbi subnuclei showed the largest displacement during the observation period. However, all cells from dmHbi migrated greater distances than the cells from vHb during the time observed (P < 0.05) (Fig. 2h, Additional file 1: Video 1). Caudal and ventral to the dmHbi, the cluster of cells pertaining to the vHb nucleus had cells that did not migrate relative to their initial position. The cells in vHb extended axons caudally (Fig. 3g, cyan), while axon bundles from the dmHbi nuclei at 48 hpf projected towards the vicinity of the nMLF, i.e. more rostrally when compared to vHb projections at this stage (Fig. 3g, magenta). All these projections form the habenula form the fasciculus retroflexus. At 96 hpf, axons from the FR became more compact, with the Hb nuclei being densely packed with *spon1b:GFP* positive cells [17] (Figs. 2, 3).

### Methods

#### Animal care and maintenance

Adult zebrafish were housed in a controlled multi-tank recirculating water system (Aquaneering Inc.) on a 14 h light–10 h dark cycle, at 27 ± 1 °C, according to standard protocols [18]. All protocols were approved by the Institutional Animal Care and Use Committee of Universidad de los Andes (code C.FUA_15-029).

#### LSFM imaging

Embryos were screened for GFP signal under a fluorescent stereoscope (Nikon AZ100M). Positive embryos were dechorionated and mounted into fluorinated ethylene propylene tubes in 0.1% agarose with tricaine (150 mg/L). Briefly, our custom-built LSFM uses a 488 nm laser, a 10 × /0.25 objective lens (Leica) to produce a light sheet of ~ 1.5 μm. A 40 × /0.8 W water objective lens (Nikon) with a bandpass filter HQ525/50M (Chroma) and a Neo camera (ANDOR) make the detection path. Temperature and aeration were maintained in the specimen chamber with a recirculating water bath. Stacks were taken at 200 ms exposure (power on sample 1.8–2.0 mW), every 1.0 μm.

#### Image processing

Image processing was performed in FiJi ImageJ [19]. Brightness and contrast were adjusted for better visibility. Transmitted and fluorescence images were overlaid for anatomical context. A color coded MIP Fiji macro developed by Beretta et al. [13] was applied to code depth with color. Stacks were aligned with the FiJi plugin Linear Stack Alignment with SIFT [20]. Aligned MIPs were exported to bitplane Imaris 8.2.0 for single cell tracking. Cells were modelled as 6 μm spheres and tracked manually. 3D reconstructions were done in Imaris to measure the distance between Hb subnuclei. Schematic drawings of embryos were made using Inkscape.

#### Data analysis

Statistical analyses to compare dynamics of vHb and dHb cells were performed in Graphpad Prism 7. The Mann–Whitney U test was applied to assay differences between the two Hb subnuclei.

## Limitations

- GFP detection requires expression, maturation and accumulation of the protein in cells, so the times reported here are delayed with respect to expression. Results with anti-*spon1b* fluorescence in situ hybridization (FISH) might be slightly different, especially in regions with abundance of projections and no cell somas.
- *spon1b* mRNA expression using in situ hybridization (ISH) was not used, since the correspondence of *spon1b* expression using ISH and the transgenic line was previously confirmed [4].
- The function of F-spondin remains elusive, and additional experiments that block the protein and test expression in individual cells should be considered.

## Supplementary information

**Additional file 1: Video 1.** Displacement of cells in the habenula subnuclei from 48 to 75.5 hpf. Time-lapse imaging allowed tracking individual cells from the dorsal habenula (dHb) and ventral habenula (vHb) subnuclei, showing that dHb cells exhibit a greater displacement (measured as displacement squared) when compared to the vHb, as represented by the displacement color code. The video is a progression of MIPs from a depth of 250 µm. Initial time point corresponds to 48 hpf. Original stacks were cropped and aligned using the FiJi plugin Linear Stack Alignment with SIFT (Lowe, 2004). Gamma was adjusted to a value of 0.75. Anterior is left. Dorsal views.

## Data Availability

Raw datasets from exemplary developmental stages (24, 48, 72, and 96 hpf) were made publicly available in the following repository: https://figshare.com/projects/Patterns_of_spon1b_GFP_expression_during_early_zebrafish_development/72812

## Abbreviations

dHb: dorsal habenula
dmHbi: dorsomedial inferior habenula
dpf: days post-fertilization
GFP: green fluorescent protein
Hb: habenula
hpf: hours post-fertilization
LDFM: light sheet fluorescence microscopy
